# Design of multi-epitope vaccine against porcine rotavirus using computational biology and molecular dynamics simulation approaches

**DOI:** 10.1186/s12985-024-02440-9

**Published:** 2024-07-22

**Authors:** Xiaochen Zhu, Xinyuan Wang, Tingting Liu, Dongchao Zhang, Tianming Jin

**Affiliations:** 1 Tianjin Key Laboratory of Agricultural Animal Breeding and Healthy Husbandry, College of Animal Science and Veterinary Medicine, Tianjin Agricultural University, Tianjin, 300392 China; 2 Tianjin Key Laboratory of Animal Molecular Breeding and Biotechnology, Institute of Animal Science and Veterinary, Tianjin Academy of Agricultural Sciences, Tianjin, 300381 China; 3 Tianjin Engineering Technology Center of Livestock Pathogen Detection and Genetic Engineering Vaccine, College of Animal Science and Veterinary Medicine, Tianjin Agricultural University, Tianjin, 300392 China

**Keywords:** Porcine Rotavirus, Bioinformatics, Multi-epitope vaccine, Molecular dynamic simulation

## Abstract

*Porcine Rotavirus* (PoRV) is a significant pathogen affecting swine-rearing regions globally, presenting a substantial threat to the economic development of the livestock sector. At present, no specific pharmaceuticals are available for this disease, and treatment options remain exceedingly limited. This study seeks to design a multi-epitope peptide vaccine for PoRV employing bioinformatics approaches to robustly activate T-cell and B-cell immune responses. Two antigenic proteins, VP7 and VP8*, were selected from PoRV, and potential immunogenic T-cell and B-cell epitopes were predicted using immunoinformatic tools. These epitopes were further screened according to non-toxicity, antigenicity, non-allergenicity, and immunogenicity criteria. The selected epitopes were linked with linkers to form a novel multi-epitope vaccine construct, with the PADRE sequence (AKFVAAWTLKAAA) and RS09 peptide attached at the N-terminus of the designed peptide chain to enhance the vaccine’s antigenicity. Protein-protein docking of the vaccine constructs with toll-like receptors (TLR3 and TLR4) was conducted using computational methods, with the lowest energy docking results selected as the optimal predictive model. Subsequently, molecular dynamics (MD) simulation methods were employed to assess the stability of the protein vaccine constructs and TLR3 and TLR4 receptors. The results indicated that the vaccine-TLR3 and vaccine-TLR4 docking models remained stable throughout the simulation period. Additionally, the C-IMMSIM tool was utilized to determine the immunogenic triggering capability of the vaccine protein, demonstrating that the constructed vaccine protein could induce both cell-mediated and humoral immune responses, thereby playing a role in eliciting host immune responses. In conclusion, this study successfully constructed a multi-epitope vaccine against PoRV and validated the stability and efficacy of the vaccine through computational analysis. However, as the study is purely computational, experimental evaluation is required to validate the safety and immunogenicity of the newly constructed vaccine protein.

## Introduction

*Porcine Rotavirus* (PoRV), belonging to the *Rotavirus* genus within the *Reoviridae* family, is characterized by double-stranded RNA and lacks an envelope [1]. First identified in clinical samples of pig feces in 1975 [2], PoRV has emerged as a significant epidemic threat to the global swine industry, causing viral diarrhea in piglets across various swine-rearing regions. PoRV is primarily transmitted through the fecal-oral route, causing clinical symptoms in piglets such as vomiting, anorexia, dehydration, diarrhea, acid-base imbalance, and ultimately resulting in fatality [3]. Pathological changes in the small intestine are primarily caused by the proliferation of PoRV in villous epithelial cells, the destruction of these cells, and subsequent adaptive and regenerative responses. Most medium-sized and large pigs generally have latent infections without exhibiting any symptoms. Piglets are highly susceptible to infection due to their immature immune defense mechanisms, especially when they are less than four weeks old. Piglets less than 10–20 days old have a higher case fatality rate in the absence of maternal antibodies. Additionally, the case fatality rate of PoRV-infected piglets under one week of age approaches 100% [4]. Currently, there is no specific drug or treatment for this disease. Antibiotics can only mitigate secondary bacterial infections, raising concerns about antibiotic resistance [5]. Vaccination remains the primary method for preventing and controlling viral infection [6].

The complete genome sequence of *rotavirus* is approximately 18 kb in length. It comprises 11 double-stranded RNAs (dsRNA) encoding six structural proteins (VP1, VP2, VP3, VP4, VP6, and VP7) and five to six nonstructural proteins (NSP1 to NSP5/6) [1]. The genome of *rotavirus* is encapsulated by three layers of structural proteins. Proteins VP7 and VP8* compose the outer capsid and determine viral G and P types, respectively; they are the primary immunogens that can induce the body to produce neutralizing antibodies and cellular immune responses [7, 8]. The production of inactivated vaccines is complex, time-consuming, and costly. Moreover, the immune response to these vaccines is delayed, reducing their effectiveness in emergency prevention [9]. Attenuated vaccines can effectively stimulate the immune system; however, the risks of re-infection and side effects must be considered [10, 11]. Clinical evidence indicates that the risk of intussusception increases within 1 to 7 days after PoRV vaccination, especially following the first vaccination [12]. Furthermore, traditional preventive vaccines are allergenic, potentially dangerous, and require in vitro cultivation of pathogenic viruses; these factors hinder the development of such vaccines.

Studies have identified VP7 and VP8* proteins of *rotavirus* as ideal targets for vaccine development. Numerous *rotavirus* vaccine candidates currently under development focus on these outer capsid proteins [13]. VP7 is the major constituent of the outer protein and serves as the principal neutralizing antigen of *rotavirus*. It possesses a Ca2 + binding function critical for promoting virion maturation during virus replication and facilitating *rotavirus* invasion into host cells [14]. The spike protein VP4 of PoRV can stimulate the body to produce neutralizing antibodies and is considered an important target for anti-PoRV strategies [15]. The VP8* protein, generated by cleaved VP4, interacts with host receptors, initiating the infection process. It is pivotal in recognizing and binding to host cell surface carbohydrates. Moreover, the core region of VP8* encompasses nearly all antigenic epitopes of VP4, acting as a key neutralizing antigen [16]. This avoids the presence of additional antigens in other regions that may cause excessive antigen burden and increase the probability of allergic reactions [17 ~ 19]. Similar to the full-length spike protein, VP8* can stimulate the production of neutralizing antibodies in the body, thereby activating immune protection.

Indeed, epitope-based vaccines offer promising alternatives to traditional vaccines. Leveraging immunoinformatics and computational biology, researchers have developed vaccine candidates against a wide array of pathogens. These approaches enable the precise identification and design of antigenic epitopes, potentially enhancing vaccine efficacy and safety profiles [20 ~ 22]. These tools have played a crucial role in assisting with epitope screening, sequencing, and design, thereby enhancing the specificity, safety, and stability of vaccines. In recent years, epidemiological investigations of PoRV have revealed the dominance of the G9 genotype. This genotype has the potential to spread rapidly worldwide and is highly homologous to the VP7 gene of the human G9 strain [23, 24]. The emergence of the G9P [23] strain, a genotype of the *rotavirus*, has been observed in various regions, including Taiwan, South Korea, and Thailand, among others. Notably, it appears to be prevalent among diarrheal piglets in northern Thailand [25 - 27]. It is noteworthy that the genotype has demonstrated recombination events between human and pig strains, as well as virus transmission between pigs and humans [28, 29]. In this regard, it holds significant implications for both public health and veterinary health.

This study focused on the Group A G9P [23] strain, utilizing immunoinformatic techniques to develop an immunogenic multi-epitope vaccine candidate derived from the VP7 and VP8* sequences of PoRV’s outer proteins. The vaccine incorporates epitopes recognized by helper T lymphocytes (HTLs), cytotoxic T lymphocytes (CTLs), and B cells (BLs), interconnected with linkers and supplemented with gene adjuvants to bolster the immunogenic response. Various structural verification tools were employed to scrutinize the vaccine’s architecture, while its antigenicity and allergenicity were evaluated. Subsequently, the constructed vaccine underwent molecular docking and molecular dynamics simulations with TLR receptors. This comprehensive computational immune simulation analysis yielded a novel and highly effective candidate vaccine against PoRV.

## Methodology

### Protein sequence retrieval

**Fig. 1:**
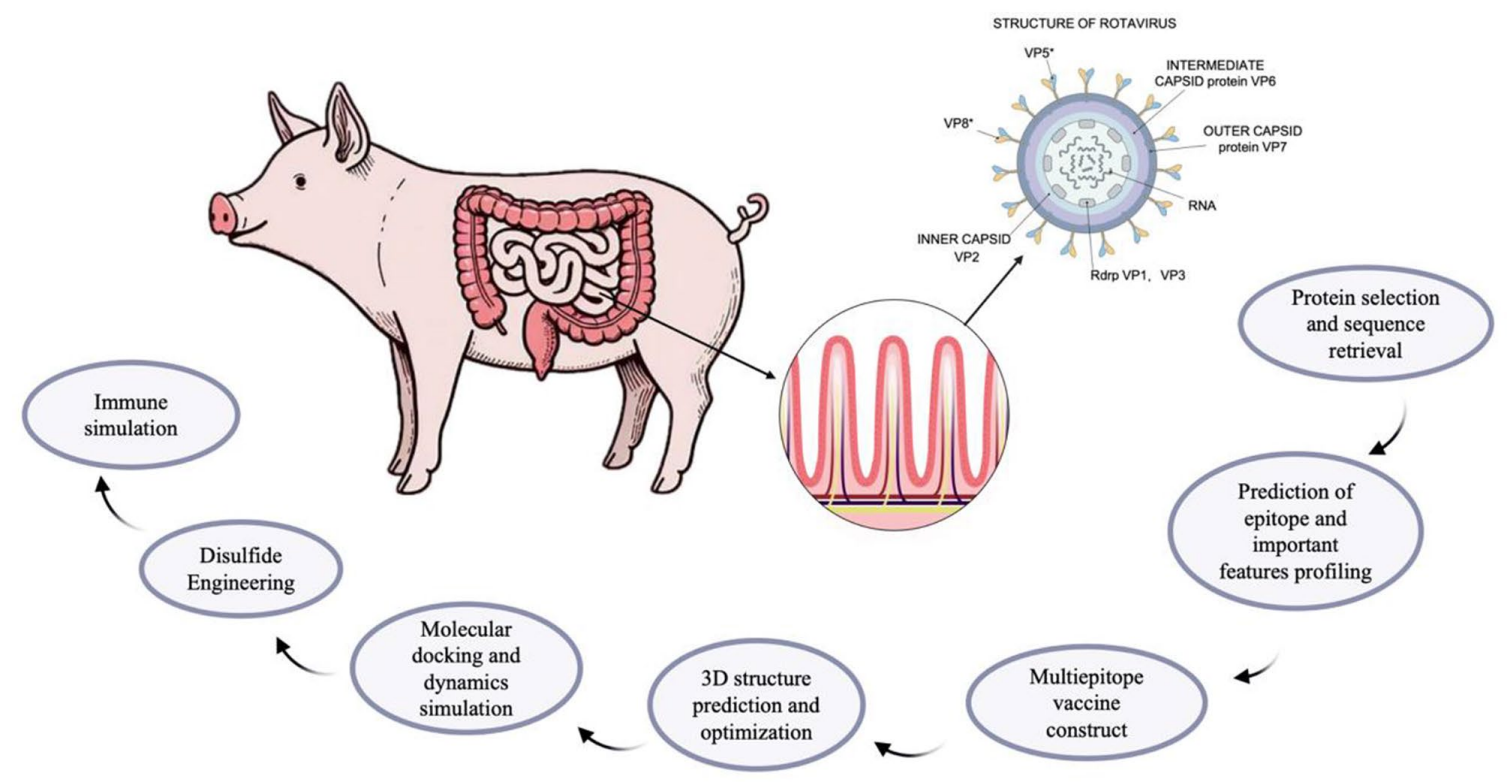
The working flow chart of this study. The method first retrieves the protein sequence and then predicts epitope and vaccine structure. To evaluate stability and binding affinity, TLR receptors were docked using a Light Dock server. Finally, the immune simulation study was carried out

The PoRV VP7 protein (accession number: MH137265.1) and VP8 protein (accession number: MH898990.1) were retrieved from the NCBI database (https://www.ncbi.nlm.nih.gov/, accessed on 2 Sep 2023) in FASTA format. Subsequently, a suitable vaccine candidate against PoRV was designed using the strategy outlined in Fig. 1.

### T lymphocyte epitope prediction

In this study, MHCI epitopes of target proteins were predicted using NetMHCI software (https://services.healthtech.dtu.dk/services/NetMHC-4.0/, accessed on 5 Sep 2023) [30] and the MHC-I binding prediction tool available on the IEDB server (http://tools.iedb.org/mhci/, accessed on 5 Sep 2023) [31]. NetMHCI software selected pigs as the species, and SLA-10,401 and SLA-20,401 as the alleles. The IEDB server also selected pigs as the species, with SLA-10,101 and SLA-10,202 as the alleles, and NetMHCpan 4.1 EL (recommended epitope predictor-2023.09) as the prediction method. When HTLs engage with phagocytes, they release cytokines that trigger the proliferation and differentiation of other immune cells, including BLs, CTLs, and macrophages. The study used EpiTOP software (http://www.pharmfac.net/EpiTOP, accessed on 7 Sep 2023) [32] and IEDB’s MHC-II binding prediction tool (http://tools.iedb.org/mhcii/, accessed on 7 Sep 2023) to predict the MHCII epitopes of the protein. EpiTOP software selected DRB1*0101 as the allele, and pIC50 = 6.3 as the threshold. The pIC50 = 6.3 indicates that this MHCII epitope may be a strong immunogen. The higher the pIC50 value, the greater the binding affinity between the epitope and MHCII, making it easier to trigger the immune response. The IEDB server selected human HLA-DR as the allele, with NetMHCIIpan 4.1 EL (recommended epitope predictor-2023.09) as the prediction method. Peptides with overlapping alleles and higher scores from each software were selected as candidates for further epitope screening.

### Linear B lymphocyte epitope prediction

B cells play a crucial role in humoral immunity and produce large amounts of antibodies that bind directly to antigens to provide long-lasting immunity. The study employed the IEDB antibody epitope prediction tool (http://tools.iedb.org/bcell/, accessed on 8 Sep 2023)and the ABCpred linear epitope prediction method (https://webs.iiitd.edu.in/raghava/abcpred/ABC_submission.html, accessed on 8 Sep 2023) [33] to predict the linear B cell epitopes of the target protein. Peptides exhibiting overlapping and high scores from each software were chosen as candidates for subsequent epitope screening. This approach facilitates the identification of protein regions likely to elicit an antibody response.

### B‑and T‑cell epitopes important features profiling

Vaxijen v2.0 (http://www.ddg-pharmfac.net/vaxijen/VaxiJen/VaxiJen.html, accessed on 28 Sep 2023) [34] is a pioneering server for forecasting protective antigens and subunit vaccines, eliminating the need for sequence alignment. Its accuracy ranges between 70% and 89%. In this study, viruses were designated as the target organisms, with an antigenic threshold of 0.5. AllerCatPro (https://allercatpro.bii.a-star.edu.sg/allergy/index.html, accessed on 14 Sep 2023) [35] was used to assess the allergenicity of epitopes. AllerCatPro compares input amino acid sequences against an allergen dataset to evaluate their allergenic potential. ToxinPred (https://webs.iiitd.edu.in/raghava/toxinpred/design.php, accessed on 14 Sep 2023) [36], a tool that leverages diverse peptide properties, was utilized to forecast epitope toxicity with an accuracy of approximately 90%.

### Design of multiepitope vaccine construct

Epitopes are chosen based on their superior antigenicity scores, as well as their allergenicity and toxicity analyses. AAY, GPGPG, and KK are used to link MHC-I, MHC-II, and B cell epitopes, respectively. AAY is a linker used to connect MHC-I epitopes, improving the hydrophilicity and stability of the epitope and enhancing its affinity for MHC-I molecules [37]. GPGPG serves as a linker for connecting MHC-II epitopes, enhancing the safety, specificity, and flexibility of the epitope by enabling it to adapt to the binding groove of the MHC-II molecules [38]. KK is a linker used to join B cell epitopes, thereby boosting the antigenicity and immunogenicity of the epitope [37]. EAAAK serves as a linker for connecting different types of epitopes. Through the EAAAK linker, the RS09 peptide and the PADRE sequence were introduced at the N-terminus of the vaccine construct as genetic adjuvants. The RS09 peptide functions as an LPS peptide mimetic, acting as an artificial TLR4 agonist. By mimicking the binding of LPS to TLR4, it enhances the immune response and boosts antibody production [39, 40]. The PADRE sequence (AKFVAAWTLKAAA) is a universal helper T cell epitope capable of binding to various HLA-DR molecules, activating CD4 + T cell immune responses, and stimulating the secretion of various cytokines [41]. Linking the PADRE sequence to antigenic epitopes enhance the vaccine’s immunogenicity and stability [42].

### Evaluation of the vaccine candidate’s antigenicity and physiochemical properties

The vaccine candidates underwent evaluation for antigenicity and allergenicity using the Vaxijen server v2.0 and the AllerCatPro server, respectively. Additionally, the solubility was assessed using the Protein-Sol server (*Protein-sol sequence solubility manchester.ac.uk*, accessed on 28 Sep 2023) [43]. This server calculates 35 sequence-based properties and compares them to a solubility database of *E. coli* proteins, providing a scaled solubility value between 0 and 1, with higher values indicating greater solubility compared to average soluble *E. coli* proteins. The Expasy server’s ProtParam tool (https://web.expasy.org/protparam/, accessed on 28 Sep 2023) [44] was used to assess the candidate vaccine’s physicochemical parameters.

### Secondary structure prediction and three-dimensional modeling

The SOPMA server( https://npsa-prabi.ibcp.fr/cgibin/npsa_automat.pl?page=npsa%20_sopma.html, accessed on 3 Oct 2023) [45] was employed to predict the secondary structure elements of the vaccine candidate, including alpha-helix, extended strand, beta-turn, and random coil. This information provides insights into the overall structural characteristics of the vaccine. To predict the tertiary structure of the vaccine construct, AlphaFold 2 was utilized. AlphaFold 2 represents a significant advancement in protein structure prediction by employing a novel deep-learning approach called the attention mechanism. This method enables the model to learn intricate relationships between amino acids within a protein sequence and their spatial positions in the 3D structure, leading to highly accurate predictions. AlphaFold 2 achieved unprecedented accuracy in the CASP14 competition, where it predicted the 3D structures of 100 proteins with an average global distance test (GDT) score of 92.4, indicating that the majority of its predictions closely matched the experimental structures [46]. It is the most accurate protein tertiary structure prediction model to date and can predict protein structures with atomic-level accuracy. It is especially good at predicting the structures of proteins that had no homologous structures in the Protein Data Bank [47]. The model underwent additional optimization by submitting it to the GalaxyWEB server (https://galaxy.seoklab.org/cgi-bin/submit.cgi?type=REFINE2, accessed on 5 Oct 2023) [48]. The GalaxyWEB server’s template-based modeling method identifies and refines problematic areas in the template using ab initio calculations. Structural validation was conducted using analysis from the Ramachandran plot, ERRAT plot (https://saves.mbi.ucla.edu/, accessed on 5 Oct 2023) [49, 50] and ProSA-web server (https://prosa.services.came.sbg.ac.at/prosa.php, accessed on 5 Oct 2023) [51].

### Prediction of discontinuous B cell epitopes

The ElliPro tool (http://tools.iedb.org/ellipro/, accessed on 7 Oct 2023) [52] on the IEDB server was used to analyze discontinuous B-cell epitopes. Thanks to the use of geometric fitting and protrusion analysis, ElliPro can accurately identify potential epitopes exposed on the protein surface. The PDB file of the refined vaccine tertiary structure was uploaded as the input file, and the epitope prediction parameters used the default values. The minimum score and maximum distance were set at 0.5 and 6, respectively.

### Molecular docking

TLRs can recognize pathogen-associated molecular patterns (PAMPs) and induce the expression of immune effector molecules such as cytokines and chemokines, thus initiating and regulating the synergy between innate and adaptive immunity. Therefore, their interactions with the candidate vaccine are critical. The vaccine structure and TLR3 protein, TLR4 protein were docked using a rigid protein-protein docking method, and the docking process was completed through the LightDock online server (https://server.lightdock.org/, accessed on 10 Oct 2023) [53]. The full-length structure of TLR3(UniProt: A0A0E3N2 × 7) and TLR4 (UniProt: A0A7K8I613) were obtained from the AlphaFold server [47].

LightDock is a docking framework for protein-protein, protein-peptide, and protein-DNA interactions based on the Glowworm Swarm Optimization (GSO) algorithm. GSO simulates the natural behavior of fireflies, which attract each other based on the amount of emitted light, to search for the optimal protein binding sites and configurations for rigid docking. This significantly improves the search algorithm’s efficiency. Download the complex model and use the PBDePISA online server (https://www.ebi.ac.uk/msd-srv/prot_int/cgi-bin/piserver, accessed on 12 Oct 2023) [54] to check the protein docking results. PBDePISA is an online server for the assessment and analysis of molecular docking results based on PISA. It analyzes the geometric and physical characteristics of each interface and calculates various parameters, such as the interface area, contact area, hydrogen bonds, salt bridges, disulfide bonds, etc. It also estimates the binding energy and stability of the interface based on these parameters, rather than performing qualitative tests based only on the sequence, structure, similarity, and other factors of the molecule. Finally, PyMOL is used to visualize the docking conformation [55].

Rosetta is a comprehensive software suite for modeling macromolecular structures based on the simulated annealing algorithm, which can study the structure, function, interaction, and design of biomolecules. Rosetta relax is an application of Rosetta that can perform all-atom refinement of protein structures and eliminate unreasonable conformations. The principle of Rosetta Relax is to identify the local energy minimum of a given structure through small backbone adjustments, rotamer trials, and minimization [56]. PyRosetta is a Python interface developed by the PyRosetta team led by Jeffrey J. Gray, Sergey Lyskov, and others at Johns Hopkins University to use the capabilities of the Rosetta molecular modeling suite [57]. In this study, PyRosetta Relax was used for energy minimization of the complexes obtained from molecular docking, with the number of iterations set to 500.

### Molecular dynamics simulation

To gain deeper insights into the binding dynamics and stability of the vaccine construct-TLR complex, molecular dynamics (MD) simulations were performed. Simulations were executed using Gromacs 2019.8 with the AA/L all-atom force field [58]. Using the SPC/E water model [59], a water box was constructed 1 nm away from the protein surface. This box was subsequently filled with solvent and ions to achieve system neutralization. Following this, the steepest descent method was employed to conduct 50,000 steps of energy minimization for the system. After energy minimization, the system was subjected to isothermal-isovolumetric (NVT 300 K) and isobaric (NPT 1 bar) equilibration. Finally, molecular dynamics simulations (100 ns) were performed under periodic boundary conditions with a 2 fs time integration step, and the simulated data were analyzed with a 100 ps snapshot interval. Once the simulation was concluded, the root-mean-square deviation (RMSD), solvent-accessible surface area (SASA), and hydrogen bonds (H-bonds) of the system were calculated. XMGRACE v5.1 was used to generate graphs for these analyses. All MD simulations were conducted on high-performance simulation stations running Ubuntu 16.04 LTS. In addition, the MM-PBSA.py tool in Amber24 was utilized to calculate the binding free energy of the vaccine construct-TLR complex. In the MM-PBSA analysis, 100 frames from a 100 ns simulation trajectory were taken for the calculation [60].

### Disulfide engineering

Integrating disulfide bond design into predictive models can significantly improve vaccine stability. The DbD2 server (http://cptweb.cpt.wayne.edu/DbD2/, accessed on 17 Oct 2023) [61] offers a tool for this purpose. It assesses the likelihood of disulfide bond formation between different residue pairs based on the protein’s 3D structure and the geometric criteria for disulfide bonds. By predicting whether specific residues can form stable disulfide bonds upon mutation to cysteine, this technology improves protein thermal stability within the host body.

### Codon optimization and in-silico cloning

Codon optimization of the vaccine candidates was performed using GenSmart Codon Optimization software developed by GenScript to improve expression efficiency and yield in host cells. For laboratory validation and experimental animal immunity studies, we selected humans and Sus scrofa (pig) as the primary and secondary expression hosts for vaccine optimization. During the optimization process, we specifically avoided KpnI and SalI restriction endonuclease sites. The vaccine sequences were then inserted into the pEGFP-N1 expression vector using SnapGene software.

### Immune simulation

To assess the vaccine’s immunogenicity and immune response profile, we utilized the C-IMMSIM web server available at https://kraken.iac.rm.cnr.it/C-IMMSIM/ (accessed on 22 Oct 2023) [62]. C-IMMSIM functions as a computational model of the immune system, simulating adaptive humoral and cellular responses to antigens. The interval between prime and boost vaccinations plays a pivotal role in achieving an optimal humoral response. However, analyzing the impact of each injection on peak antibody titers becomes challenging when multiple injections are administered within a short timeframe [63]. Therefore, in this investigation, the vaccine was administered every four weeks to forecast the post-vaccination immune response. The simulation was configured to 200 steps, adhering to default parameters except for the injection time step, which occurred at step 1, 84 (equivalent to 4 weeks), and 168 (equivalent to 8 weeks).

## Results

### Superior epitope selection

T-cell and B-cell epitopes of capsid proteins VP7 and VP8 were identified using NetMHCI, IEDB, EpiTOP, and ABCpred. This resulted in the selection of four T-cell epitopes (two each for MHC-I and MHC-II) and four B-cell epitopes. Vaxijen v2.0 was used to predict the immunogenicity of an antigen. A predicted result greater than 0.5 indicates antigenicity, whereas a result less than 0.5 indicates non-antigenicity [34]. All selected epitopes were antigenic. Toxinpred software was used to analyze the toxicity of the selected epitopes [36]. All candidate epitopes were non-toxic and non-mutant. AllerCatPro predicted that all candidate epitopes are non-allergenic. An amphiphilicity value greater than 0 indicates that the peptide has strong amphiphilicity and can be stably dispersed in a water environment, while a value less than 0 indicates that the peptide may be biased towards hydrophilicity or hydrophobicity. Positive hydrophilicity signifies that the peptide is highly hydrophilic, well-soluble in water, and more stable during storage and transportation. All selected peptides were hydrophilic. The main features and annotations of the *in silico* predicted epitopes are listed in Table 1 ~ 3.

**Table 1:** Predicted antigenicity of T-cell epitopes

Epitope type	Protein	EPITOPE	Alleles		VaxiJen Score
MHC-I	VP7	NPMDITLYY	SLA-1*0202	SLA-20,401	1.4099
	VP8	QTGYAPVNW	SLA-1*0201	SLA-20,401	1.6625
MHC-II	VP7	FKEYTNIASFSIDPQ	HLA-DRB1*01:01	DRB1*0101	0.8670
	VP8	PGPFAQTGYAPVNWG	HLA-DRB1*01:03	DRB1*0101	1.6925

**Table 2:** Predicted antigenicity of B-cell epitopes

Epitope type	protein	ABCpred	VaxiJen Score	IEDB	VaxiJen Score
B-cell	VP7	ASTQIGDTEWKDTLSQ	1.0083	TEASTQIGDTEWKDT	1.4857
	VP7	CPLNTQTLGIGCITTN	1.3445	LNTQTLGI	0.6402
	VP8	IQIIGSEKTQNVTINP	1.0443	LSNSYTVDLSDEIQIIGSEKTQNVTI	0.7761
	VP8	AQTGYAPVNWGPGETN	1.5885	GPFAQTGYAPVNWGPGETNDSTTVEPVLDGPYQPTTFNP	0.9433

**Table 3:** Allergenicity, toxicity of and physiochemical properties selected epitopes

EPITOPE	Allergenicity	Toxicity	Amphipathicity	Hydrophilicity
NPMDITLYY	Non-allergen	Non-Toxic	-0.74	0.67
QTGYAPVNW	Non-allergen	Non-Toxic	-0.86	0.78
FKEYTNIASFSIDPQ	Non-allergen	Non-Toxic	-0.12	0.80
PGPFAQTGYAPVNWG	Non-allergen	Non-Toxic	-0.71	0.47
ASTQIGDTEWKDTLSQ	Non-allergen	Non-Toxic	0.27	0.94
CPLNTQTLGIGCITTN	Non-allergen	Non-Toxic	-0.64	0.62
IQIIGSEKTQNVTINP	Non-allergen	Non-Toxic	-0.15	0.88
AQTGYAPVNWGPGETN	Non-allergen	Non-Toxic	-0.34	0.69

### The construction of multi-epitope vaccine

**Fig. 2:**
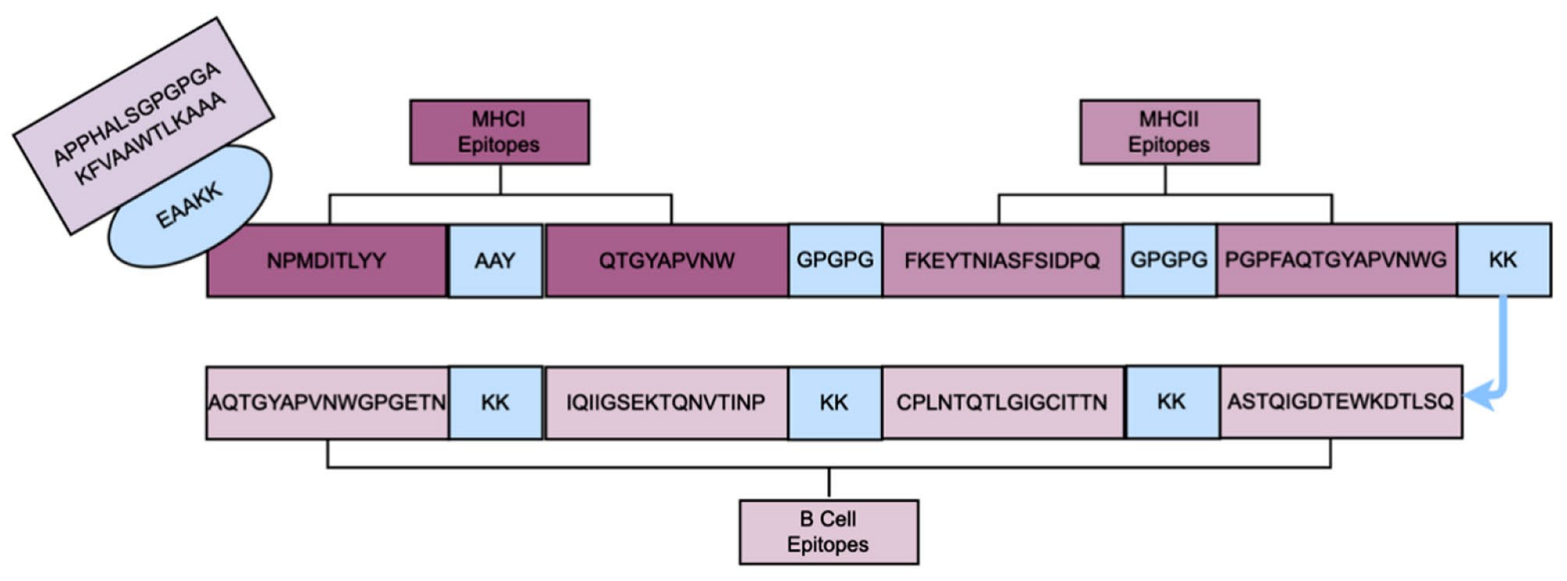
Illustration of the final vaccine building block. The diagram shows the arrangement of the different epitopes, with the adjuvant at the N-terminal and the linker shown in blue

The vaccine construct incorporated the PADRE sequence and the RS09 peptide as adjuvants. A series of linkers, including AAY, GPGPG, and KK, were used to connect two CTL epitopes, two HTL epitopes, and four B-cell epitopes, respectively. An EAAAK linker was used to attach the adjuvant to the N-terminus of the vaccine, and a 6×His tag was introduced at the C-terminus to verify protein expression in future experiments. Figure 2 shows a schematic diagram of the finalized multi-epitope vaccine, which comprises 163 amino acids.

### Evaluation of the vaccine candidate’s antigenicity, allergenicity, solubility, and physiochemical properties

**Fig. 3:**
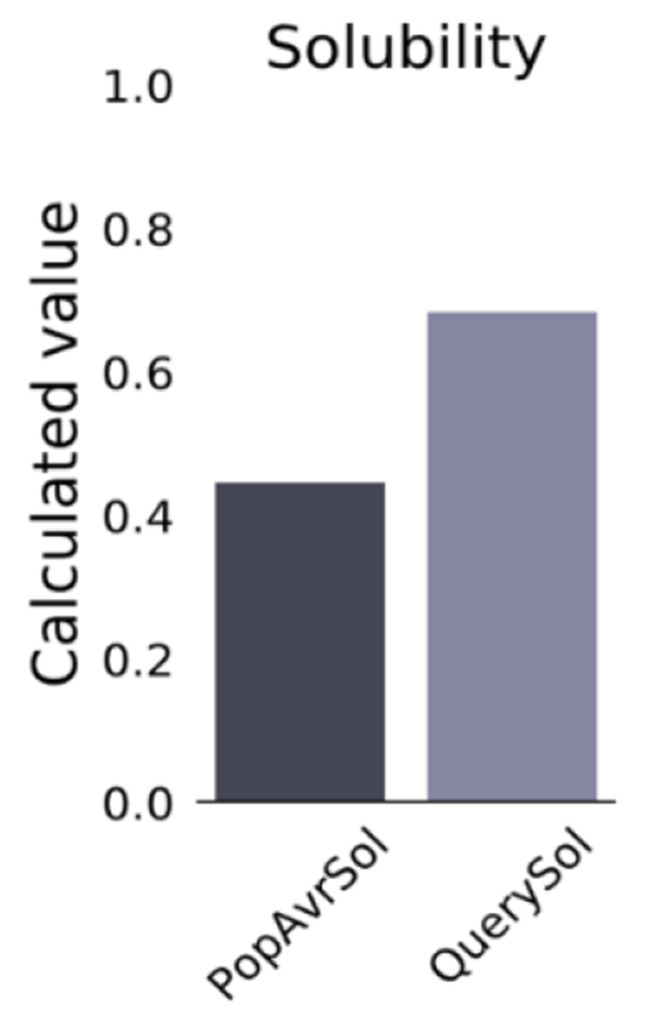
Solubility of multi-epitope vaccines predicted by ProteinSol server. The solubility of the vaccine was 0.628, which was soluble protein

The Vaxijen v2.0 server was utilized to predict the immunogenicity of the vaccine, yielding a value of 0.9318. A value above the threshold of 0.5 indicates that the vaccine can stimulate the immune system to produce an immune response. The vaccine candidate was predicted to be non-allergenic by AllerCatPro. The ToxinPred server predicted that the vaccine structure was non-toxic. The ProtParam tool was employed to evaluate the physicochemical properties of the vaccine candidate. Positive values were assigned to hydrophilic amino acids and negative values to hydrophobic amino acids. The grand average of hydropathy (GRAVY) for the designed vaccine structure is -0.472. The results indicated that the vaccine exhibited high hydrophilicity, contributing to the stability and effectiveness of the vaccine. The calculated instability index (II) is 16.05. The smaller the instability index, the more stable the protein. With 40 as the critical value, this protein is stable. With 40 as the critical value, this protein is stable. The aliphatic index was 59.45. The aliphatic index reflects the relative value of fatty side chain amino acid content. The fat index is high, the protein is easy to form a tight spherical structure, and the heat stability is high. The estimated half-life of the vaccine in yeast (in vivo) and *E. coli* (in vivo) is over 20 h and 10 h, respectively. The theoretical pI was 9.19, and the comprehensive evaluation showed that the vaccine was stable after expression. Additionally, the Protein-Sol server forecasted a solubility of 0.628 for the vaccine (refer to Fig. 3), while experimental data from the PopAvrSol dataset yielded a solubility value of 0.45. The predicted solubility value exceeds 0.45, indicating that the protein has high solubility.

### Secondary structure prediction

**Fig. 4:**
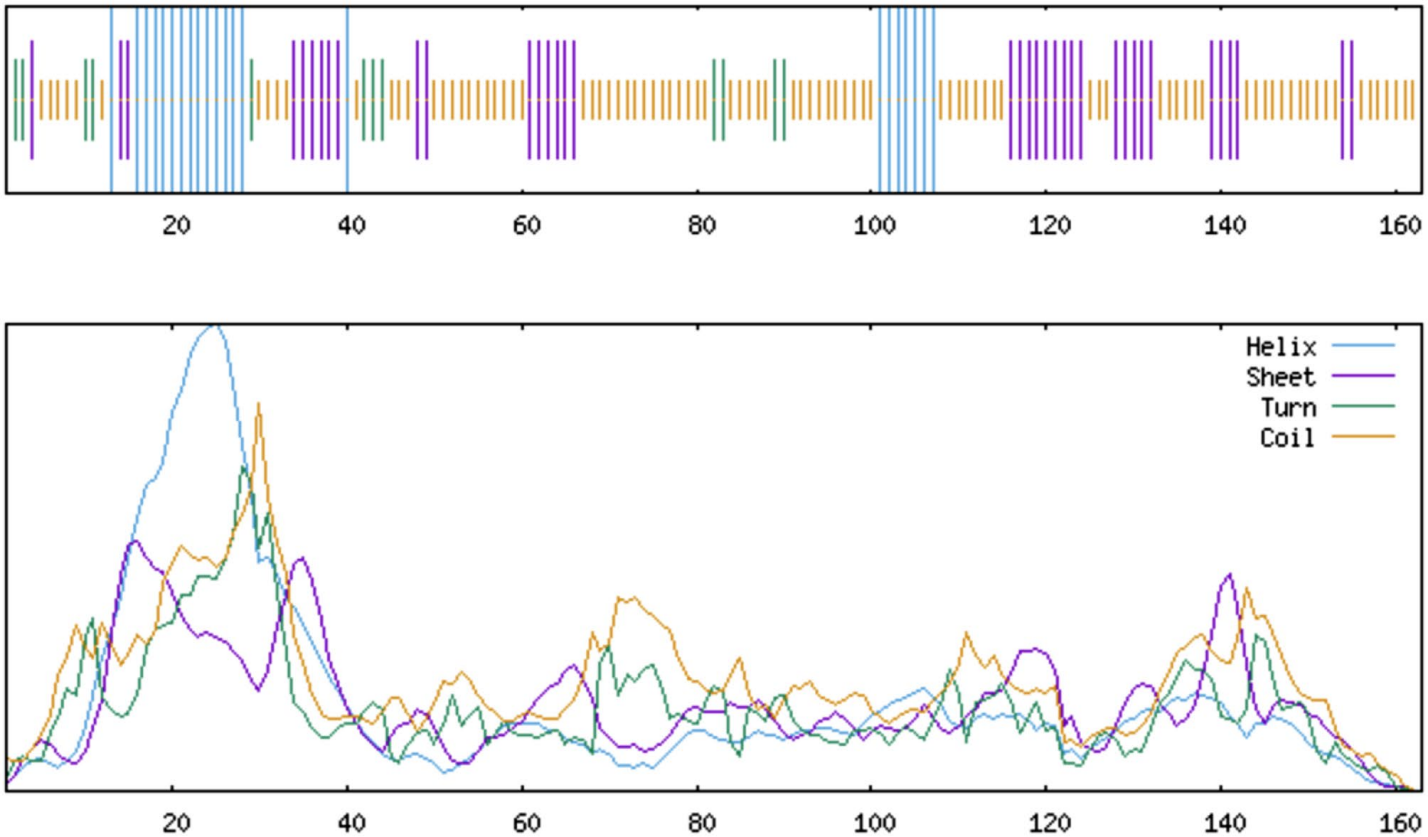
Illustration of the secondary structure of a vaccine. The vaccine consisted of 25 (15.34%) alpha-helices, 40 (24.54%) extended strands, 11 (6.75%) beta-turns, and 87 (53.37%) random coils

The secondary structure predicted by SOPMA online software is shown in Fig. 4. The analysis of the secondary structure showed that the vaccine consists of 25 (15.34%) alpha-helices, 40 (24.54%) extended strands, 11 (6.75%) beta-turns, and 87 (53.37%) random coils. The vaccine structure contains a small number of alpha-helices and beta-turns and a large number of random curls. This suggests that the vaccine may not have a fixed structure and has a high degree of flexibility, which helps it bind to the immune system in the host. Additionally, because no specific folding is required, the vaccine protein is more easily expressed and more stable during production.

### Prediction and optimization of vaccine tertiary structure

**Fig. 5:**
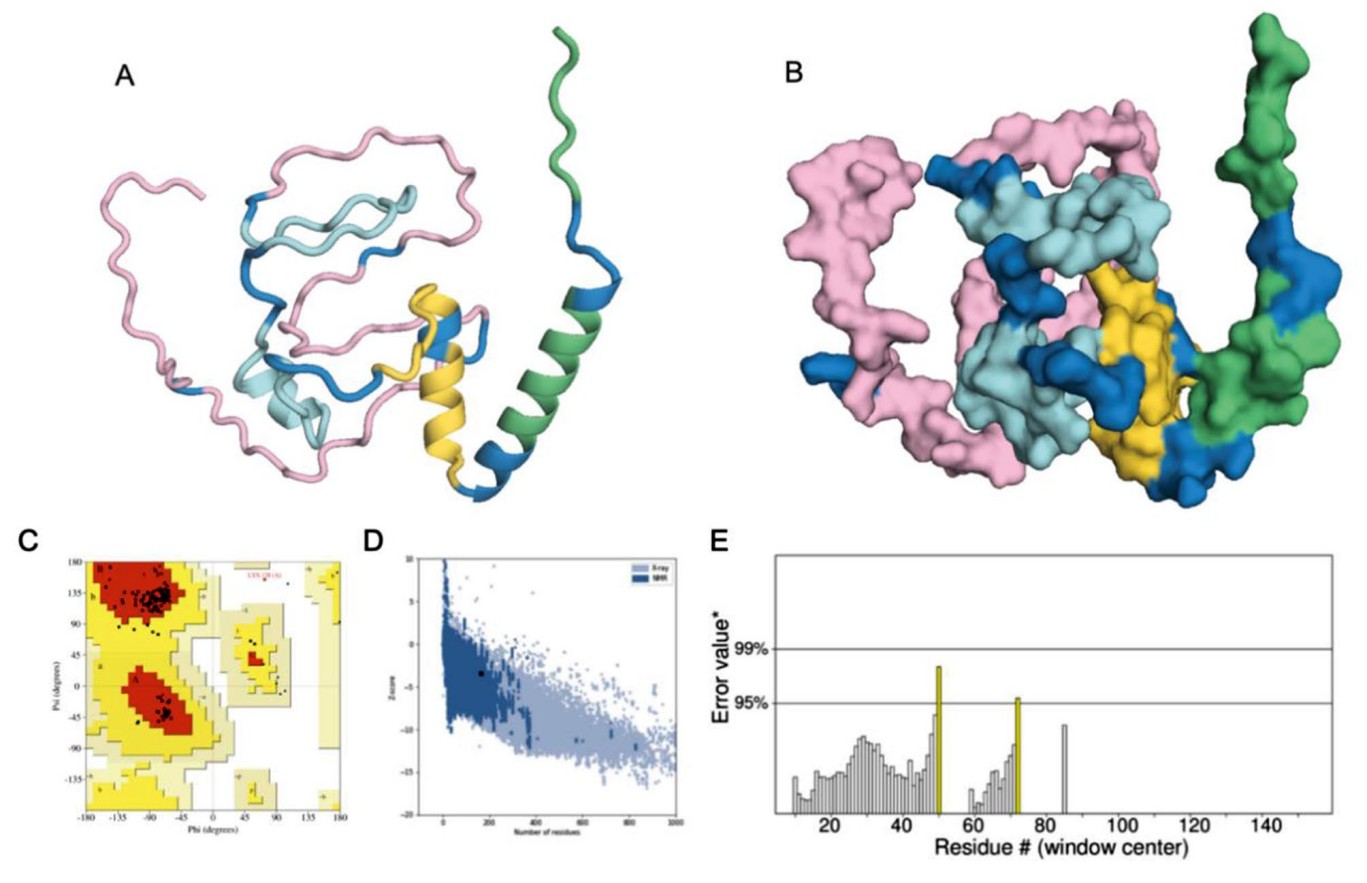
Tertiary structure and structural validation of the designed vaccine candidate. (**A**) The cartoon format of vaccine candidates (Linker: blue, HTL epitopes: yellow, adjuvant: green, CTL epitopes: epitopes, B-cell epitopes: pink), (**B**) The surface view of vaccine candidates, the color scheme of the vaccine construct is identical to A, (**C**) Ramachandran Plot, (**D**) ProSA model quality, and (**D**) ERRAT plot of the vaccine construct

The tertiary structure of the vaccine was modeled using the AlphaFold2 web server. AlphaFold2 predicts the confidence of each residue coordinate using the Local Distance Difference Test (pLDDT) for each residue. In the range of 0 to 100, a higher pLDDT indicates higher confidence in the predicted coordinates [64]. The software generated 5 structures and conducted a comprehensive comparison of the pLDDT scores of each structure, selecting Model 1 with the highest confidence as the final prediction result (see Fig. 5A, B). The tertiary structure model of the vaccine was refined using the GalaxyWEB server, and the best structure was chosen based on a quality assessment. The Ramachandran plot analysis revealed that out of 163 residues, 92.7% were situated in the most favored region, 6.5% in the additional allowed region, and only one residue (Lys 128) was in the disallowed region of the final model structure (refer to Fig. 5C). Subsequently, the quality and potential errors of the vaccine tertiary structure model were assessed. The Z-score obtained from the ProSA-web server was − 3.39 (refer to Fig. 5D), and the ERRAT plot score for the vaccine structure model was 96.429 (refer to Fig. 5E). These results collectively indicate that the overall quality of the model is good.

### Prediction of discontinuous B cell epitopes

**Fig. 6:**
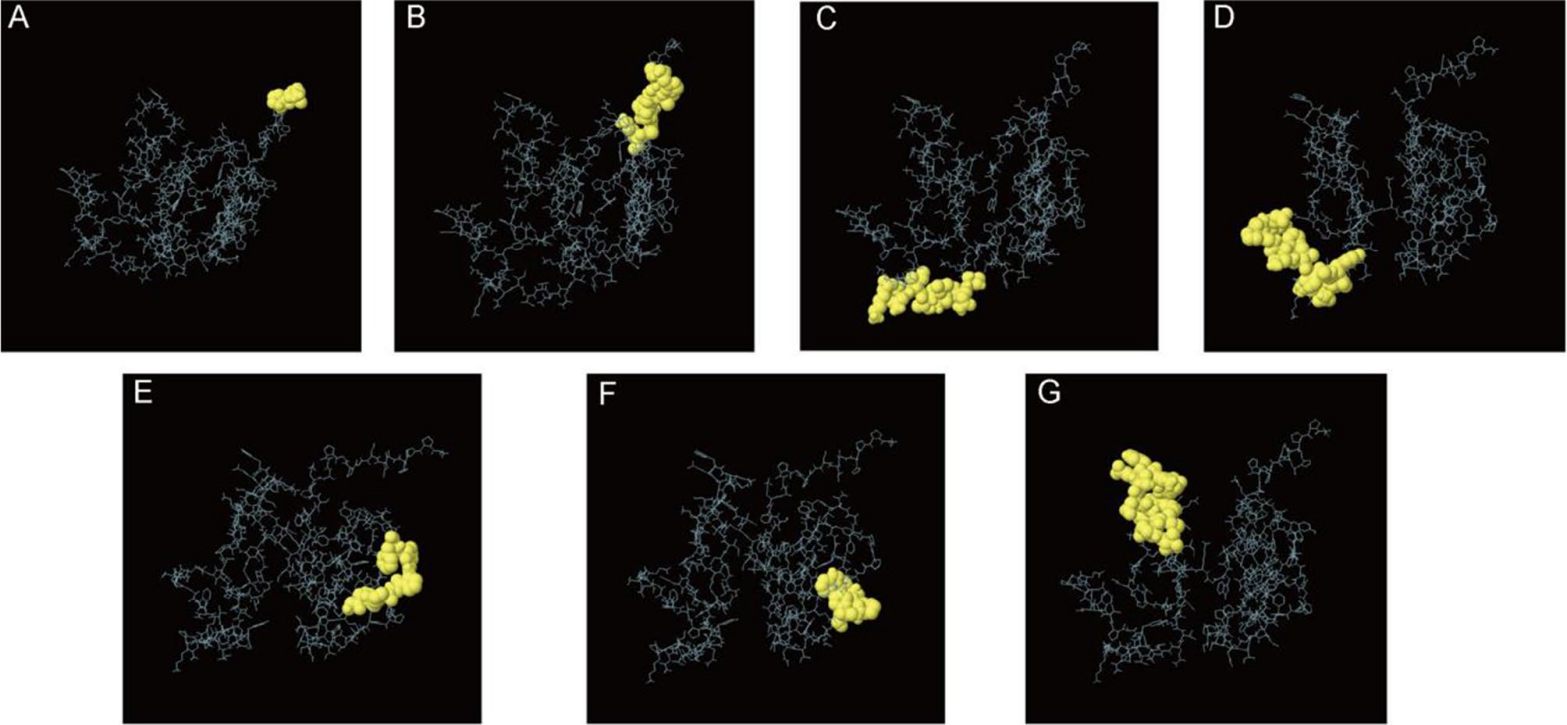
Predicting discontinuous B-cell epitopes in vaccines (**A** ~ **G**). The yellow part indicates discontinuous B-cell epitopes. (**A**) 3 residues with a score of 0.988, (**B**) 11 residues with a score of 0.843, (**C**) 9 residues with a score of 0.806, (**D**) 17 residues with a score of 0.754, (**E**) 9 residues with a score of 0.626, (**F**) 7 residues with a score of 0.613, (**G**) 15 residues with a score of 0.609

The Ellipro tool predicts seven discontinuous B-cell epitopes in the vaccine structure, as shown in Fig. 6. The details and scores of the B-cell epitopes are described in Table 4.

**Table 4:** Prediction of discontinuous B-cell epitopes in vaccines

No.	Residues	Number of residues	Score
1	A: A1, A: P2, A: P3	3	0.988
2	A: H4, A: A5, A: L6, A: S7, A: G8, A: P9, A: G10, A: P11, A: G12, A: A13, A: K14	11	0.843
3	A: T138, A: Q139, A: N140, A: V141, A: T142, A: I143, A: N144, A: P145, A: K146	9	0.806
4	A: K147, A: A148, A: Q149, A: T150, A: G151, A: Y152, A: A153, A: P154, A: V155, A: N156, A: W157, A: G158, A: P159, A: G160, A: E161, A: T162, A: N163	17	0.754
5	A: G72, A: P73, A: G74, A: P75, A: G76, A: P77, A: G78, A: P79, A: F80	9	0.626
6	A: W51, A: G52, A: P53, A: G54, A: P55, A: G56, A: F57	7	0.613
7	A: T96, A: Q97, A: I98, A: G99, A: D100, A: T101, A: E102, A: W103, A: K104, A: D105, A: T106, A: L107, A: S108, A: Q109, A: K110	15	0.609

### Molecular docking analysis

**Fig. 7:**
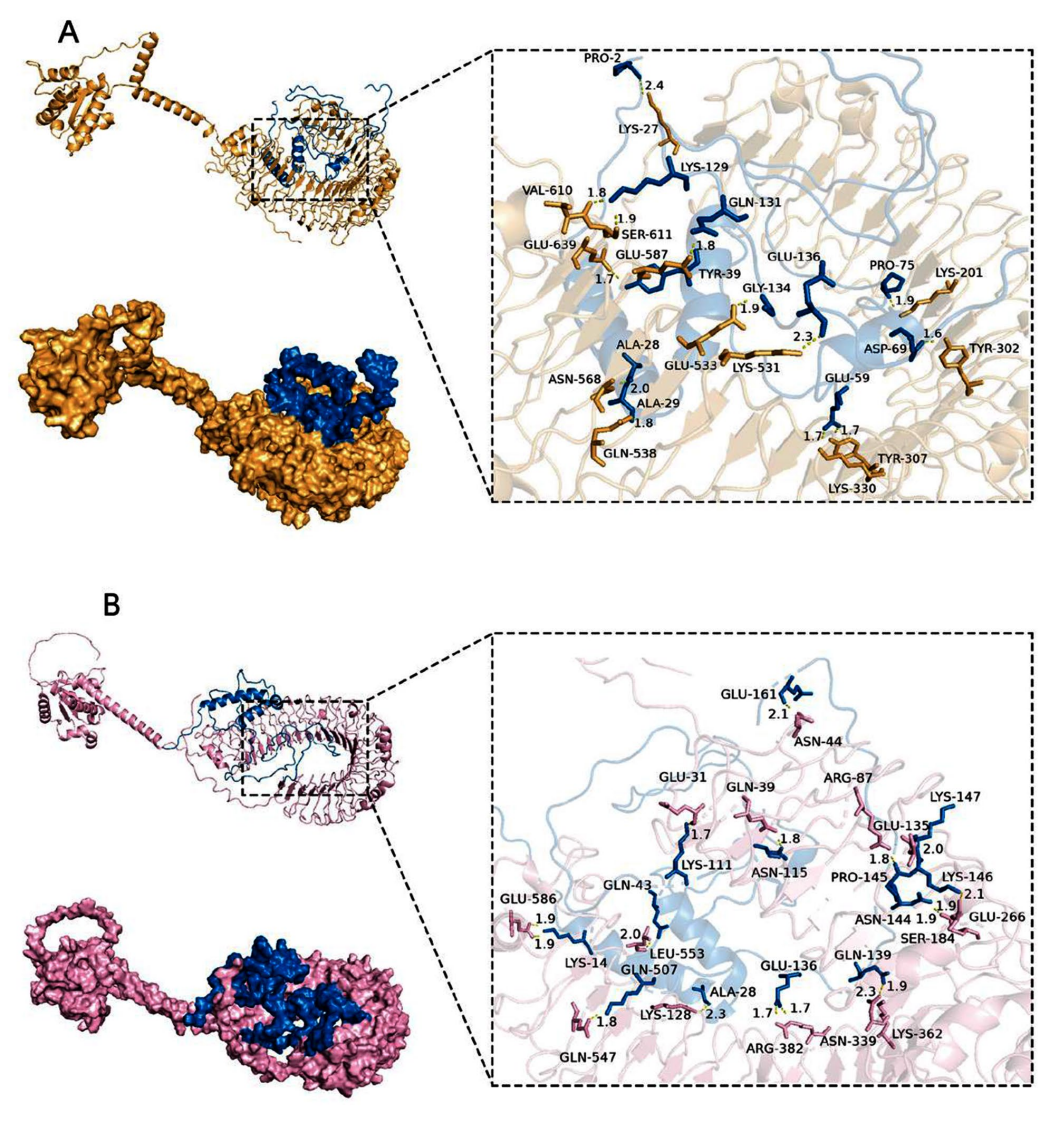
The docked complex of TLR with the vaccine, The H-bonds are shown in the inset. (**A**) The docked complex of TLR3 (yellow) with the vaccine, (**B**) The docked complex of TLR4 (pink) with the vaccine

**Fig. 8:**
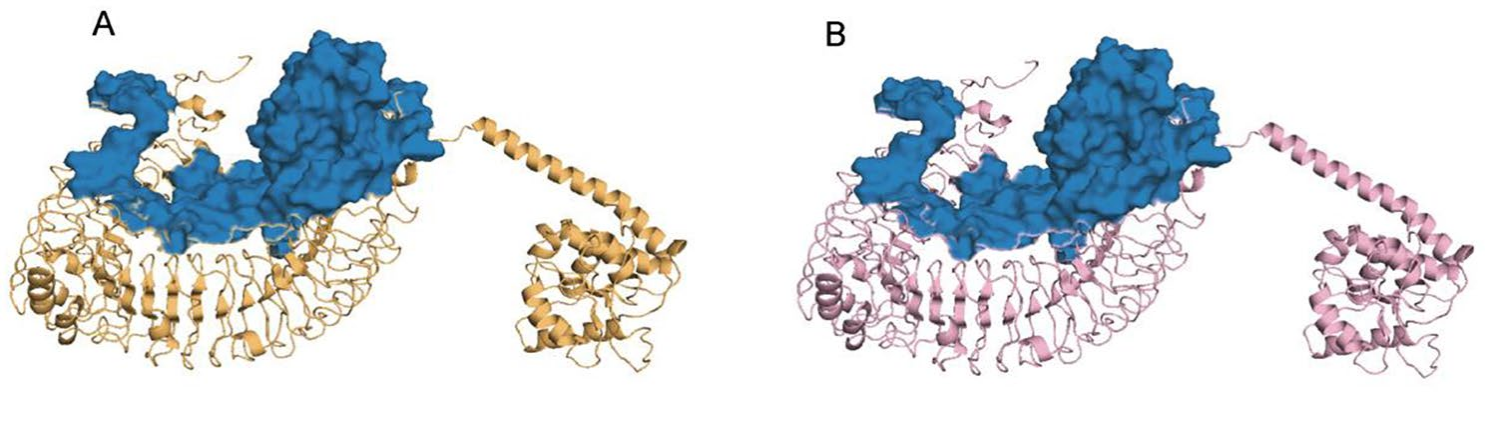
Rosetta relax optimized structure (the color scheme of the complexes is identical to Fig. 7). (**A**) The optimized complex of TLR3 with the vaccine, (**B**) The optimized complex of TLR4 with the vaccine

The LightDock server was used to perform protein docking between the vaccine and TLR3 and TLR4 after optimizing and verifying the vaccine’s tertiary structure. In LightDock’s output, the min-top file contains all the optimal solutions for the docking process and is arranged in order of energy from lowest to highest. The low-energy protein docking model better approximates the actual situation of protein interactions in living organisms and can reduce the complexity of the calculation. Therefore, we used the PBDePISA server to examine and analyze the lowest energy complex 1 (Fig. 7) and used it for subsequent analysis. The vaccine-TLR3 complex formed a total of 13 H-bonds, and the vaccine-TLR4 complex formed 17 H-bonds. Details of the docking are shown in Table 5–6. For structures treated to minimize energy, see Fig. 8A, B.

**Table 5:** The distances between the hydrogen bonding residues in the vaccine-TLR3 complex are shown

Sl. No.	TLR 3		Distance (Å)	Vaccine	
	Residue name	Residue number		Residue name	Residue number
1	LYS	27	2.41	PRO	2
2	ASN	568	1.95	ALA	28
3	GLN	538	1.81	ALA	29
4	LYS	330	1.72	GLU	59
5	TYR	307	1.66	GLU	59
6	TYR	302	1.63	ASP	69
7	LYS	201	1.86	PRO	75
8	LYS	531	2.29	GLU	136
9	GLU	639	1.67	TYR	39
10	SER	611	1.88	LYS	129
11	VAL	610	1.84	LYS	129
12	GLU	587	1.82	GLN	131
13	GLU	533	1.88	GLY	134

**Table 6:** The distances between the hydrogen bonding residues in the vaccine-TLR4 complex are shown

Sl. No.	TLR4		Distance (Å)	Vaccine	
	Residue name	Residue number		Residue name	Residue number
1	GLN	507	2.26	ALA	28
2	GLN	39	1.84	ASN	115
3	ARG	382	1.72	GLU	136
4	ARG	382	1.75	GLU	136
5	LYS	362	2.31	GLN	139
6	ASN	339	1.86	GLN	139
7	ARG	87	1.79	PRO	145
8	ASN	44	2.10	GLU	161
9	GLU	586	1.88	LYS	14
10	GLU	586	1.89	LYS	14
11	LEU	553	2.01	GLN	43
12	GLU	31	1.71	LYS	111
13	GLN	547	1.81	LYS	128
14	GLU	266	1.90	ASN	144
15	SER	184	1.85	LYS	146
16	SER	184	2.13	LYS	146
17	GLU	135	2.05	LYS	147

### Molecular dynamics simulation

**Fig. 9:**
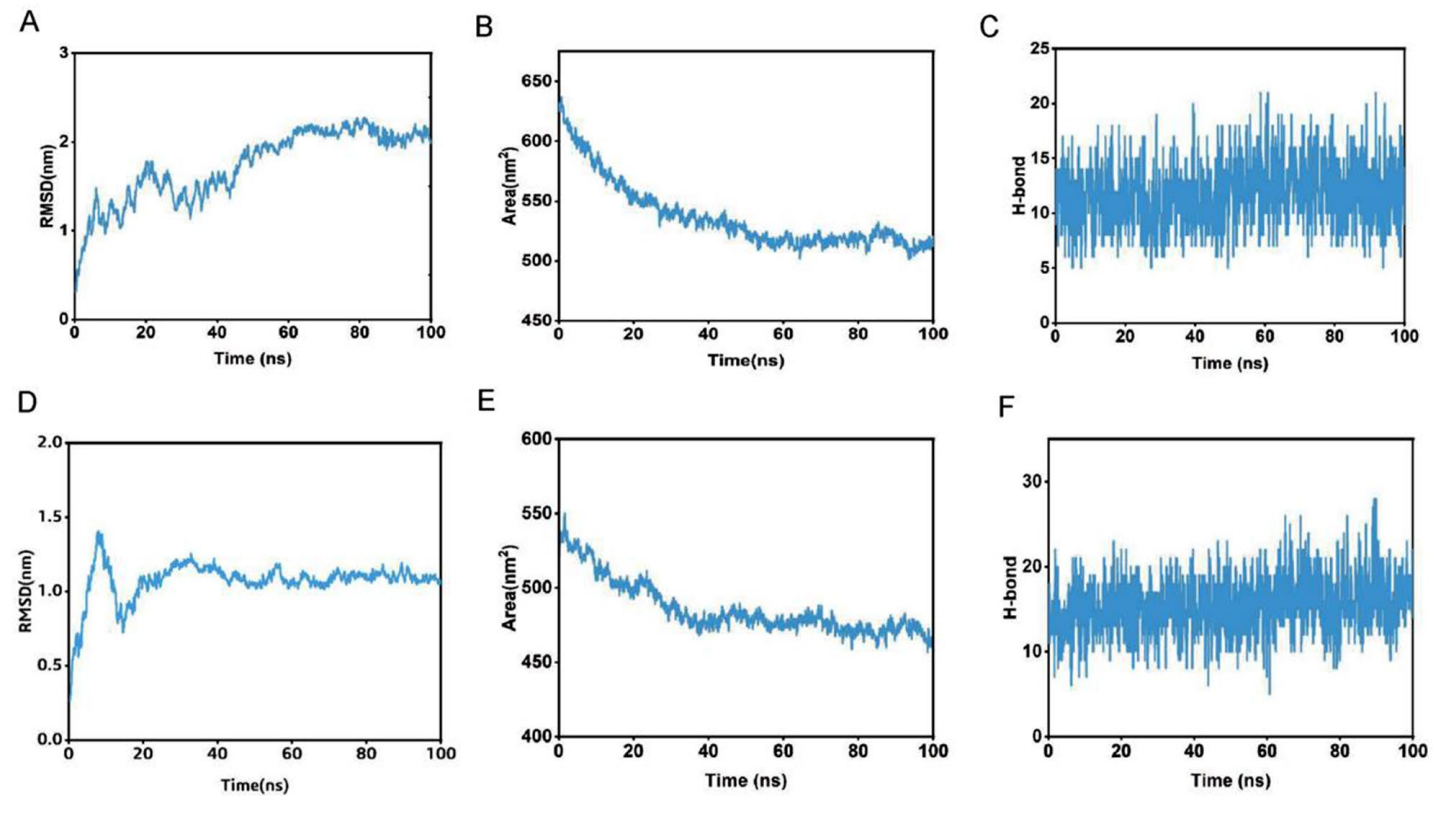
The MD simulation results. (**A**) RMSD of the TLR3-vaccine backbone, (**B**) SASA values during the simulation of TLR3-vaccine, (**C**) The hydrogen bond plot of constructed vaccine and TLR3 docked complex, (**D**) RMSD of the TLR4-vaccine backbone, (**E**) SASA values during the simulation of TLR4-vaccine, (**F**) H-bonds between the vaccine construct and TLR4

**Fig. 10:**
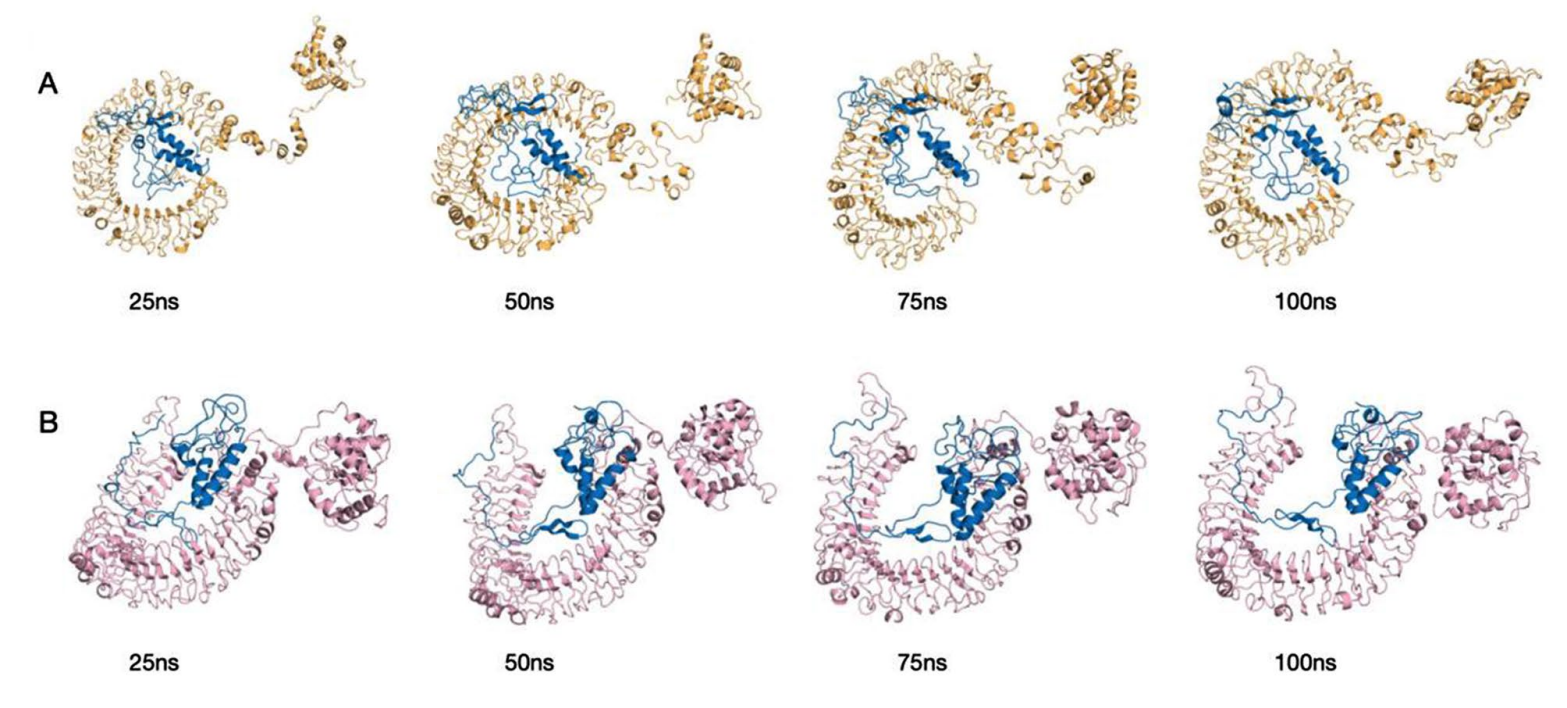
Snapshot of the simulated trajectory (at 25,50,75,100 ns). (**A**) Simulation trajectory snapshot of TLR3-vaccine, (**B**) Simulation trajectory snapshot of TLR4-vaccine

**Fig. 11:**
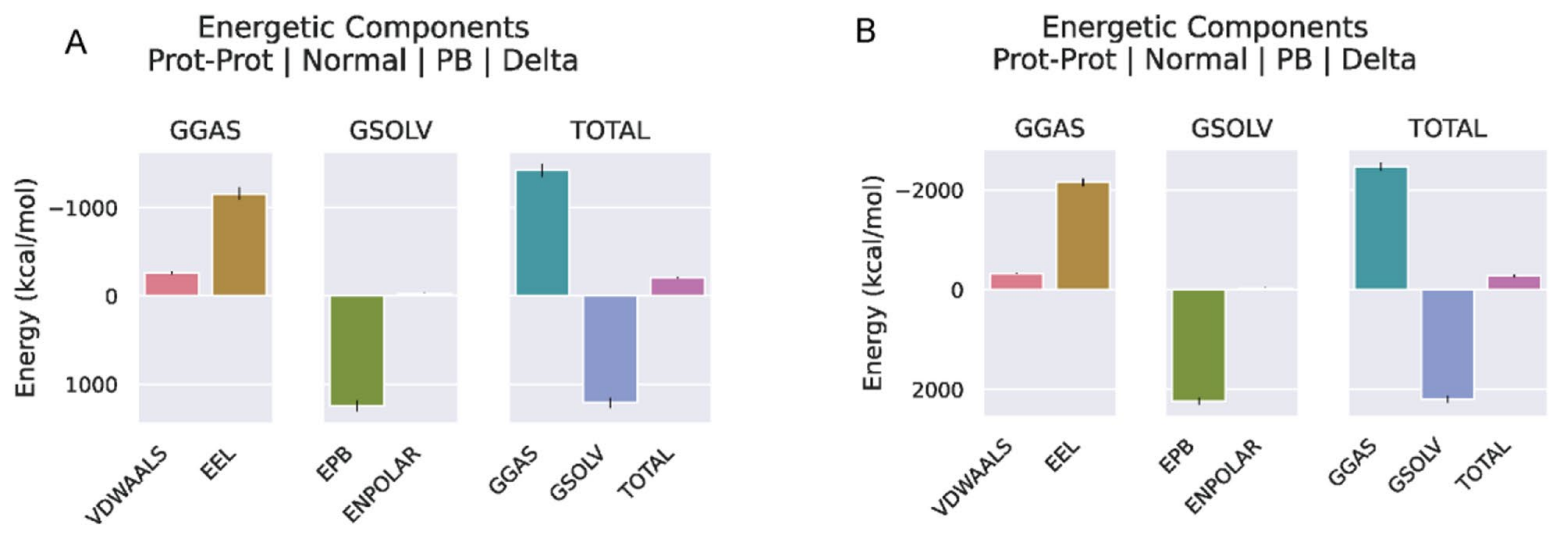
Schematic diagram of binding energy decomposition, different parameters of estimated energy are indicated using different colors. (**A**) Free binding energy estimation of TLR3-vaccine, (**B**) Free binding energy estimation of TLR4-vaccine

To analyze the stability of the TLR3, TLR4, and vaccine complexes, a 100 ns molecular dynamics simulation was performed for each complex. RMSD profile, SASA and h bond graph of the main chain residues were generated. It can be observed from the RMSD plot that the structural deviation of the TLR3-vaccine complex stabilized after 50 ns, the maximum value in the simulation was 2.27 nm at 81 ns and the average RMSD value after 50 ns was 2.07 nm. In terms of RMSD analysis, the TLR4-vaccine complex showed higher stability than the TLR3-vaccine complex. A larger RMSD value of the TLR4-vaccine complex was observed between 0 and 15 ns. However, after 30 ns of simulation, the TLR4-vaccine complex remained stable, and no drastic changes in the RMSD spectrum were observed for the simulated system. The RMSD averaged over the entire simulation was 1.07 nm, and the average after 30 ns was 1.10 nm(see Fig. 9A, D). The same trend can be seen in the SASA plot as in the RMSD plot. The decrease in the SASA value may be due to the compression of the protein structure and closer binding between the components, ultimately forming a more stable interaction. The TLR4-vaccine complex was more compact than the TLR3-vaccine complex (see Fig. 9B, E). The calculation of the hydrogen bonds between the complexes during the simulation process illustrated the stability of the complexes’ interfaces. The number of hydrogen-bonding interactions of the TLR3-vaccine complexes remained on average at 11, and those of TLR4-vaccine at 15. The lack of large fluctuations in the hydrogen bonds indicated that the binding position had not changed significantly (see Fig. 9C, F). Overall, the complex structure of the vaccine binding to TLR3 and TLR4 was stable. The trajectory snapshots in the simulation is shown in Fig. 10. Finally, the binding free energies of the docking results were calculated using MM-PBSA for TLR3-vaccine and TLR4-vaccine as -209.45 kJ/mol and − 279.83 kJ/mol. These results illustrate thermodynamically that the structures of the complexes are stable, and details are shown in Table 7; Fig. 11.

**Table 7:** Combine the details of the free energy in units are kcal/mol

parameters	VDWAALS	EEL	EPB	ENPOLAR	GGAS	GSOLV	TOTAL
Vaccine– TLR-3	-262.81	-1160.47	1245.14	-31.31	-1423.28	1213.83	-209.45
Vaccine– TLR-4	-322.67	-2151.45	2230.69	-36.40	-2474.12	2194.28	-279.83

### Disulfide engineering of the vaccine construct

**Fig. 12:**
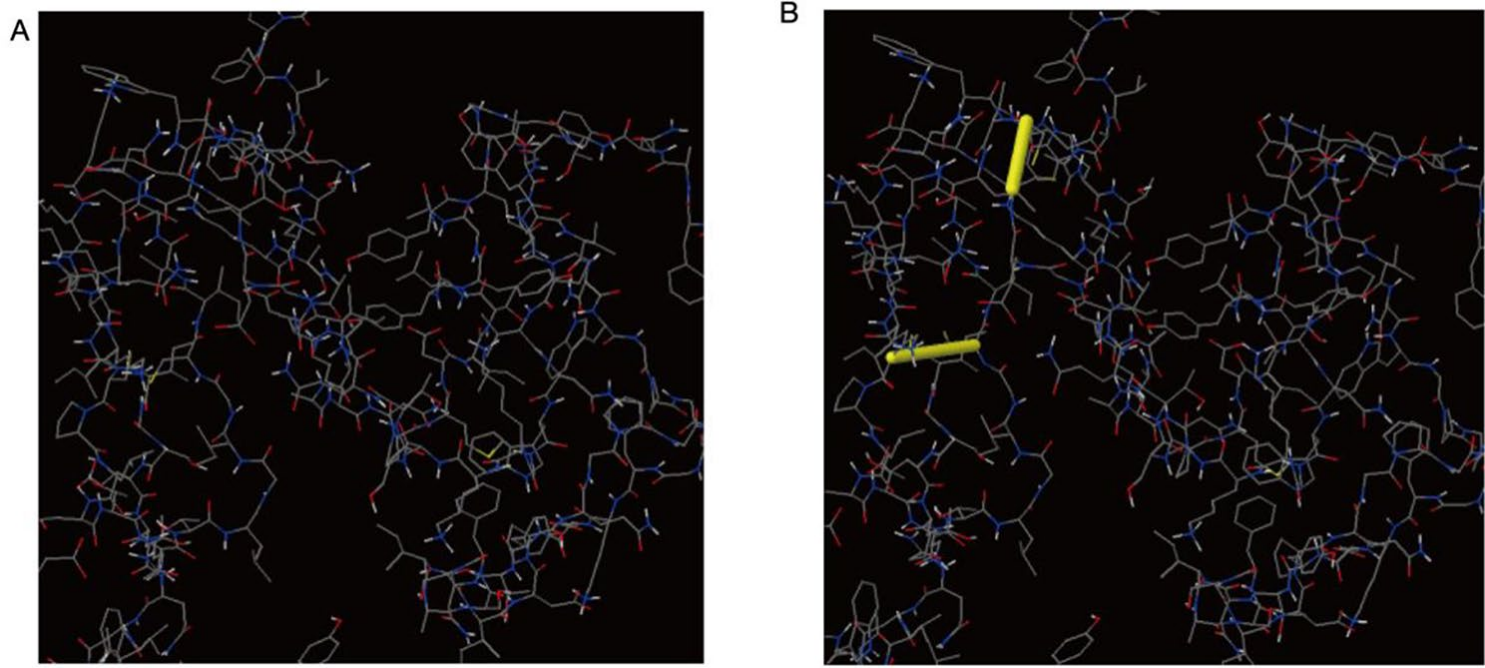
Disulfide engineering of the vaccine construct. (**A**) Original 3D model, (**B**) Mutant model with a disulfide bond

The DbD2 server identifies a total of six amino acid pairs that may form disulfide bonds and displays various parameters. χ3 represents the geometric configuration of the disulfide bond. The configuration of the disulfide bond with an extreme angle deviates from the optimal state, which may affect the disulfide bond’s stability. Disulfide bonds with high bond energy are prone to hydrolysis or reduction, affecting their stability. In the residue screening, the χ3 angle is between − 87° and + 97°±5, the energy is < 4, and two pairs (Thr101-Lys104 and Cys112-Cys123) are suitable for replacement by cysteine. Mutant models are constructed by replacing these residues with cysteine (see Fig. 12).

### Codon optimization and in-silico cloning

**Fig. 13:**
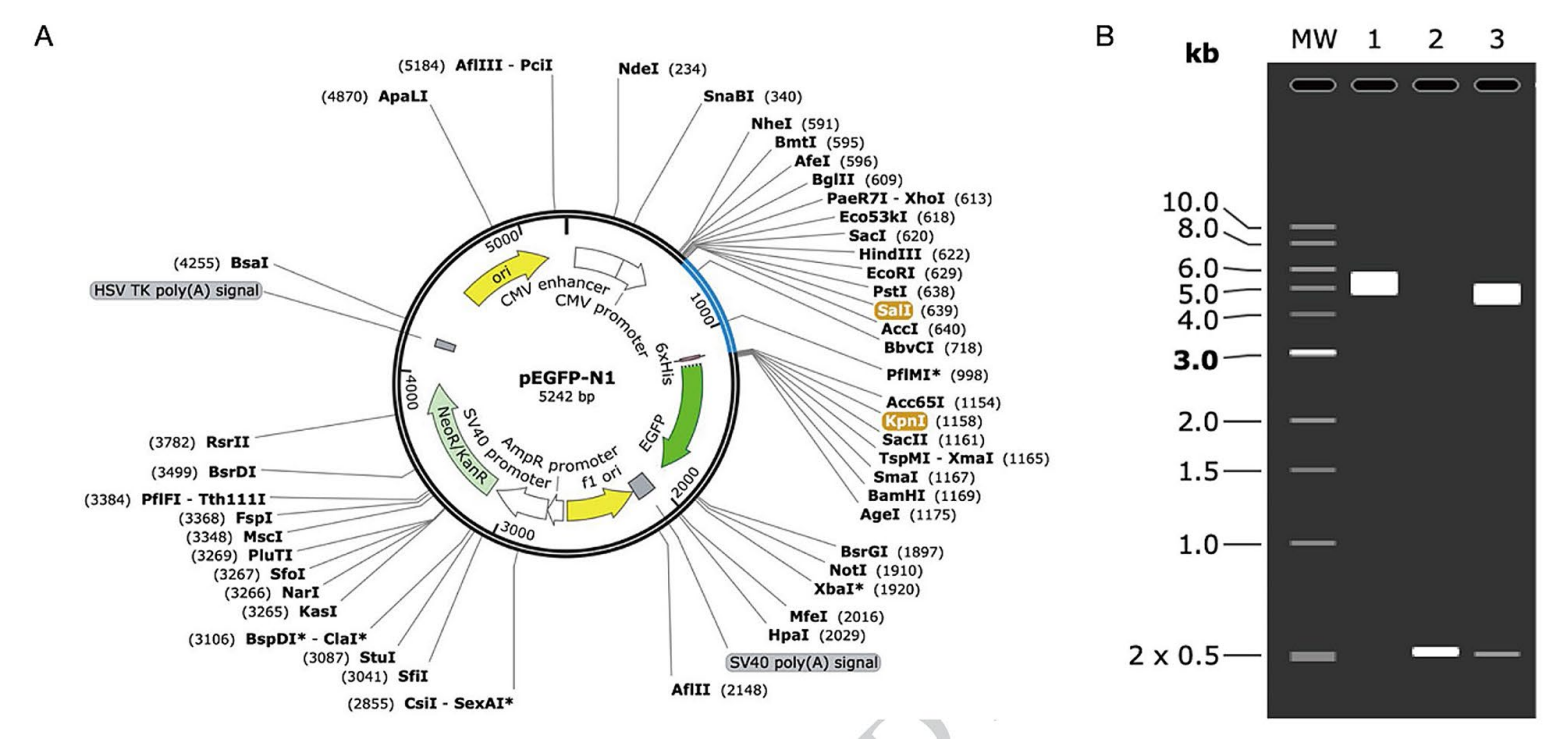
Vaccine construction in the vector and virtual digestion by double enzyme. (**A**) The multi-epitope vaccine optimization codon (blue) was cloned into the pEGFP-N1 expression vector between the SalI and KpnI cleavage sites. (**B**) Lane 1 was the natural pEGFP-N1 vector, Lane 2 was the insertion (vaccine), and Lane 3 was a double-enzyme vaccine + vector

The codon sequences were optimized to enhance protein expression in host cells using the GeneScript program resulting in an increase in the Codon Adaptation Index (CAI) to 0.9. A CAI within the range of 0.8-1.0 is considered good for high expression [65]. The revised sequence has an average GC content of 58.22%, which falls within the acceptable range, with unfavorable peaks removed. Following optimization, the improved DNA sequence was inserted between the *SalI* (639) and *KpnI* (649) restriction sites of the pEGFP-N1 vector (Fig. 13A). Moreover, the clones were virtually confirmed using the SnapGene agarose gel simulation tool. After digestion with *SalI* and *KpnI* enzymes, both the inserts (0.5 kb) and the vectors (5.2 kb) were present simultaneously. The size of the insert was consistent with the calculated molecular weight of the candidate vaccine (see Fig. 13B). Finally, the NCBI BLAST indicated no sequence similarity between the vaccine structure and the host.

### Immune response simulation

**Fig. 14:**
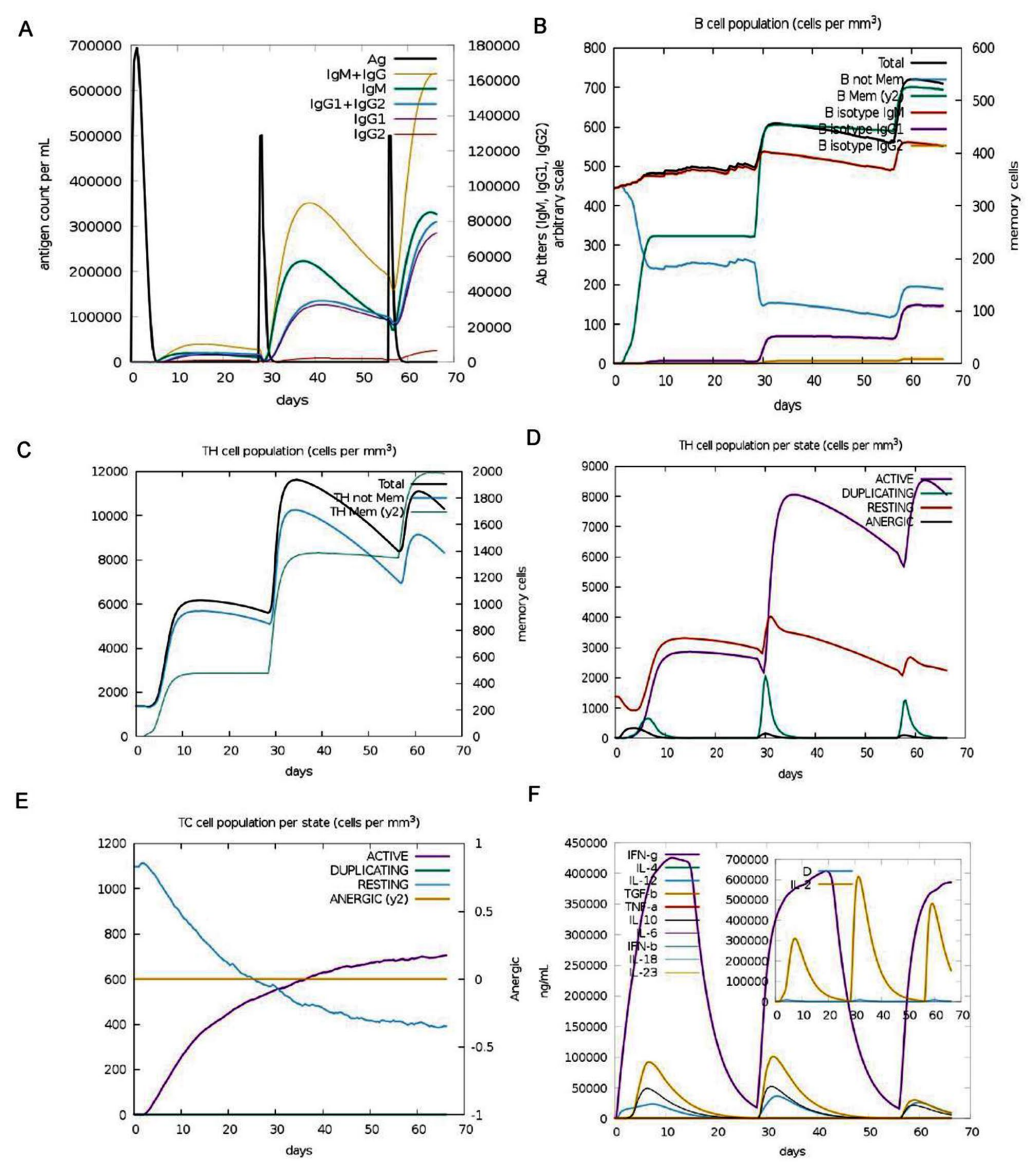
Immune simulation after receiving three doses of vaccine construct. (**A**) immunoglobulin production after vaccine administration, (**B**) B cell population after three vaccine injections, (**C**) T-helper lymphocytes, (**D**) T-helper lymphocytes per stated, (**E**) TC cells population per state, (**F**) Levels of interleukins and cytokines

The potential immunological response that the multi-epitope vaccine could induce was investigated using the C-ImmSim server [66]. The first injection of the vaccine increased the activity level of immunoglobulins, mainly causing the secretion of IgM and IgG. The second and third injections significantly further increased them (see Fig. 14A). After injection of the candidate vaccine, the number of total B cells, memory B cells, homologous IgM B cells, and homologous IgG1 B cells increased significantly. In the second and third injections, the increase was greater than in the first (see Fig. 14B). The number of active helper T cells increased after each injection of the vaccine (see Fig. 14C). After the first two injections, the number of anergic and resting helper T cells increased. However, their increase decreased after the third injection (see Fig. 14D). Elevated memory B cells and increased resting T cells may indicate that the body has been exposed to a certain pathogen or vaccine and has formed an immune memory. In cytotoxic T cells, activated cells increased, while resting cells decreased (see Fig. 14E). Vaccination also stimulated cytokine production, resulting in significant increases in IFN-γ and IL-2 (see Fig. 14F). Overall, C-ImmSim simulations predicted that the vaccine could activate immune responses.

## Discussion

Gastrointestinal infection in pigs caused by acute diarrhea is a significant problem and one of the major causes of piglet death. PoRV is considered the most important and common pathogen among these infections, frequently causing outbreaks of piglet diarrhea that seriously hinder the healthy development of the pig industry [67]. Since there is no specific drug, vaccination is the primary method to prevent and control this viral infection. Numerous studies on RV have focused on developing subunit vaccines that use immunogenic structural proteins. Tests have shown that although vaccines based on the internal capsid proteins VP2 and VP6 can stimulate the body’s immune response and produce immunoglobulins, they may perform poorly in challenge tests due to the lack of antibodies against the external capsid protein and cannot provide immune protection [68]. Therefore, VP7 and VP8 are ideal proteins for PoRV vaccine development. Recent research has confirmed this strategy. Injecting VP7 expressed in insect cells into rabbits can induce neutralizing antibodies [69]. Moreover, immunization of mice with the VP8* antigen produced high titers of IgG and protected mice from *rotavirus* challenge, resulting in a 97% reduction in fecal viral shedding compared with unimmunized controls [70].

Compared with subunit vaccines, multi-epitope vaccines can more accurately select protective epitopes while avoiding the introduction of irrelevant or harmful antigenic components, thereby improving the efficacy and safety of the vaccine. Moreover, they are easier to combine with adjuvants or carriers to enhance the immunogenicity and stability of the vaccine and reduce its side effects and cost. As reverse vaccinology advances, multi-epitope vaccines have been developed to combat SARS-CoV-2, monkeypox virus, and many other pathogens [71, 72]. There is currently no multi-epitope vaccine against PoRV. In this study, we devised a peptide-based vaccine utilizing computational biology and immunoinformatic techniques [73]. Group A G9P [23] strains were selected based on public health and veterinary health epidemiological surveys, and their VP7 and VP8 proteins were used to screen and identify B-cell and T-cell epitopes.

In the study by Sajjad et al., only the IEDB software was employed for epitope prediction. The current study mitigated false positives and enhanced prediction accuracy by integrating results from both software programs [74]. The obtained epitopes were further analyzed to select those that interact with different alleles and have antigenicity, immunogenicity, and other properties, and are non-allergenic, non-toxic, and non-mutating. Each software has different algorithms and data sets. Combining the prediction results of the two software tools can improve the accuracy and reliability of the prediction. Theoretically, using these epitopes could enable the design of vaccine candidates that elicit both humoral and cellular immunity. Linkers have been found to effectively separate epitopes in vivo and prevent the formation of linked epitopes [75, 76]. This study used AAY, GPGPG, and KK to connect MHCI, MHCII, and B-cell epitopes, and designed them into a multi-epitope vaccine. Multi-epitope vaccines are less immunogenic when used alone because their molecular weight is smaller than that of proteins, and therefore require the inclusion of adjuvants. The RS09 peptide and the PADRE sequence were introduced into the N-terminus of the vaccine structure through the EAAAK linker as genetic adjuvants to enhance the immune system’s response. Using a similar epitope-linking approach, Sajjad et al. designed a multi-epitope vaccine against the Langya henipavirus and demonstrated its potential efficacy and accessibility to the host immune system [77].

Using the ProtParam ExPASy tool to analyze the protein, we found that it had a low instability index of 16.05, indicating that the designed protein is stable. Mahnoor et al. mentioned similar experimental results, but the present study showed a lower instability index, potentially due to the use of various linkers [78]. A theoretical pI of 9.19 is assigned to the proposed vaccine, indicating it has alkaline properties similar to those of MEV candidates against canine parvovirus [20]. The aliphatic index calculations suggested that the vaccine construct is thermostable. The molecular weight of the protein is 17.1 kDa, which makes it easier to express and suitable for vaccine development [79]. Solubility affects the stability, delivery efficiency, and immune response of genetic vaccines. The high solubility of the protein enables it to enter cells and express the antigen, thus inducing a stronger immune response [80]. To prevent autoimmunity, we compared the protein sequence with the entire pig genome using NCBI BLAST and found no homology. Allergies can also pose a significant problem for vaccines. We detected no sensitization in the proteins we constructed.

The secondary structure of the vaccine contains very few α-helices and β-sheets, which is beneficial for maintaining secondary structure and spatial conformation, resulting in the constructed peptide having more coil structures [81]. AlphaFold2 was used to predict the tertiary structure in this study, and the structure with the highest confidence was selected and optimized with Galaxy WEB. I-TASSER and SWISS-MODEL software were not utilized because these tools rely on known templates or homologous sequences. These methods exhibit lower prediction accuracy and reliability for proteins without available templates or low homology [82]. According to the Ramachandran diagram analysis, the constructed vaccine exhibited 92.7% of its residues within the favorable region, with only one amino acid residue falling into the disallowed region. Additionally, the ERRAT value was 96.42, indicating a high-quality model suitable for further analysis. Ibrahim et al. conducted protein docking of the vaccine with TLR2 and TLR4 [83]. Given that TLR3 primarily recognizes double-stranded RNA, which is more pertinent to the viral genome’s structure, TLR3, TLR4, and the candidate vaccines were subjected to rigid docking in this study. Asad et al. omitted the Rosetta relax step in their study, while we included this step in ours, making our docking results align more closely with physiological conditions [84]. The results highlighted a strong affinity between the designed vaccine and the immune receptors. Molecular dynamics simulations were employed to assess the dynamics and stability of the TLR3-vaccine and TLR4-vaccine docking complexes. Analysis of the RMSD, SASA, and H-bond diagrams from the simulation results indicated stable complexes throughout the simulation period.

The designed vaccine exhibited a CAI value of 0.9 and a GC content of 58.22%, both of which fall within the optimal range. This, coupled with its high expression level in the host body, further underscores its potential effectiveness. Immune simulation results also aligned well with experimental or clinical data, indicating the vaccine’s ability to stimulate a robust immune response effectively. Taken together, these findings presented in the study collectively demonstrate the efficacy of the vaccine candidate.

## Conclusion

In this study, multiple bioinformatics techniques were used to design a multi-epitope vaccine against *porcine rotavirus* (PoRV), which causes infections in pig herds worldwide and is co-circulating with humans in many places. A vaccine is the only effective way to target PoRV infection. The vaccine combines selected antigenic, non-allergic, non-toxic, non-mutated epitopes through linkers and adjuvants. The vaccine has been evaluated as highly immunogenic, stable, and with good physical and chemical properties, and has been verified to have a very stable interaction with toll-like receptors (TLRs). The results of the study could provide valuable information and guidance for the research and application of immunology. Further in vitro and in vivo trials will be conducted to verify the effectiveness and safety of the vaccine.

## Data Availability

No datasets were generated or analysed during the current study.
